# Impact of brown rice-specific γ-oryzanol on epigenetic modulation of dopamine D2 receptors in brain striatum in high-fat-diet-induced obesity in mice

**DOI:** 10.1007/s00125-017-4305-4

**Published:** 2017-05-20

**Authors:** Chisayo Kozuka, Tadashi Kaname, Chigusa Shimizu-Okabe, Chitoshi Takayama, Masato Tsutsui, Masayuki Matsushita, Keiko Abe, Hiroaki Masuzaki

**Affiliations:** 1 Division of Endocrinology, Diabetes and Metabolism, Hematology, Rheumatology (Second Department of Medicine), Graduate School of Medicine, University of the Ryukyus, 207 Uehara, Nishihara, Nakagami-gun, Okinawa 903-0215 Japan; 2 Department of Genome Medicine, National Research Institute for Child Health and Development, Tokyo, Japan; 3 Department of Molecular Anatomy, Graduate School of Medicine, University of the Ryukyus, Okinawa, Japan; 4 Department of Pharmacology, Graduate School of Medicine, University of the Ryukyus, Okinawa, Japan; 5 Department of Molecular and Cellular Physiology, Graduate School of Medicine, University of the Ryukyus, Okinawa, Japan; 6 Department of Applied Biological Chemistry, Graduate School of Agricultural and Life Sciences, The University of Tokyo, Tokyo, Japan; 7 Kanagawa Academy of Science and Technology, Kanagawa, Japan

**Keywords:** DNA methylation, Dopamine, Epigenetics, Feeding behaviour, Nutrition, Obesity, Reward, Striatum, Type 2 diabetes

## Abstract

**Aims/hypothesis:**

Overeating of dietary fats causes obesity in humans and rodents. Recent studies in humans and rodents have demonstrated that addiction to fats shares a common mechanism with addiction to alcohol, nicotine and narcotics in terms of a dysfunction of brain reward systems. It has been highlighted that a high-fat diet (HFD) attenuates dopamine D2 receptor (D2R) signalling in the striatum, a pivotal regulator of the brain reward system, resulting in hedonic overeating. We previously reported that the brown rice-specific bioactive constituent γ-oryzanol attenuated the preference for an HFD via hypothalamic control. We therefore explored the possibility that γ-oryzanol would modulate functioning of the brain reward system in mice.

**Methods:**

Male C57BL/6J mice fed an HFD were orally treated with γ-oryzanol, and striatal levels of molecules involved in D2R signalling were evaluated. The impact of γ-oryzanol on DNA methylation of the D2R promoter and subsequent changes in preferences for dietary fat was examined. In addition, the effects of 5-aza-2′-deoxycytidine, a potent inhibitor of DNA methyltransferases (DNMTs), on food preference, D2R signalling and the levels of DNMTs in the striatum were investigated. The inhibitory effects of γ-oryzanol on the activity of DNMTs were enzymatically evaluated in vitro.

**Results:**

In striatum from mice fed an HFD, the production of D2Rs was decreased via an increase in DNA methylation of the promoter region of the D2R. Oral administration of γ-oryzanol decreased the expression and activity of DNMTs, thereby restoring the level of D2Rs in the striatum. Pharmacological inhibition of DNMTs by 5-aza-2′-deoxycytidine also ameliorated the preference for dietary fat. Consistent with these findings, enzymatic in vitro assays demonstrated that γ-oryzanol inhibited the activity of DNMTs.

**Conclusions/interpretation:**

We demonstrated that γ-oryzanol ameliorates HFD-induced DNA hypermethylation of the promoter region of D2R in the striatum of mice. Our experimental paradigm highlights γ-oryzanol as a promising antiobesity substance with the distinct property of being a novel epigenetic modulator.

**Electronic supplementary material:**

The online version of this article (doi:10.1007/s00125-017-4305-4) contains peer-reviewed but unedited supplementary material, which is available to authorised users.

## Introduction

Overeating in obese individuals shares, at least partly, common mechanisms with addiction to alcohol, nicotine and narcotics [1]. As well as the hypothalamic and hormonal regulation of appetite, the brain reward system, in particular dopamine receptor signalling, is closely related to addictive or hedonic feeding behaviour [2]. A previous study in rats showed that knockdown of the striatal dopamine D2 receptor (D2R) by lentivirus-mediated short hairpin interfering RNA rapidly induced addiction-like reward deficits and compulsion-like food seeking [3]. Because of the reduced D2R density, the dorsal striatum is less responsive to food reward compared with lean control groups in obese humans and rodents [3–5]. In accordance with this notion, the *Taq*IA allele of the *ANKK1* gene locus (encoding DRD2/ankyrin repeat and kinase domain containing 1), which decreases striatal D2R production, is associated with an obese phenotype in humans [6], while the effects of weight loss after bariatric surgery are associated with elevated striatal D2R density [7]. These data strongly suggest the importance of striatal D2R as a novel therapeutic target for the treatment of obesity. However, some drugs that were developed that acted on the brain reward system caused considerable adverse effects, including serious psychiatric problems, resulting in their eventual withdrawal from clinics [8].

Epigenetic modifications are critical not only for development and differentiation, but also because they arise as a result of environmental changes, including in diet and lifestyle [9]. DNA methylation is a chief epigenetic event for the stability of gene expression [9]. In rats, maternal exposure to a high-fat diet (HFD) intergenerationally alters DNA methylation within the central reward system in the offspring, leading to overconsumption of HFD by the pups [10]. In particular, DNA methyltransferases (DNMTs) play critical roles in the regulation of both feeding behaviour and physical activity [10, 11], suggesting that DNMTs could be promising therapeutic targets for the therapy of obesity–diabetes syndrome. Importantly, some natural food-derived substances, including caffeic acid and epigallocatechin, are known to act as DNMT inhibitors [12, 13].

We have recently shown that the bioactive, brown rice-specific component γ-oryzanol, a mixture of ferulic acid ester and several phytosterols, attenuates the preference for dietary fat via a decrease in hypothalamic endoplasmic reticulum (ER) stress [14]. In mice and rabbits, orally administered γ-oryzanol was rapidly absorbed from the intestine and distributed mainly to the brain [15, 16]. Taking these findings together, natural food-derived products acting on the central nervous system could be an alternative to safely ameliorate impaired feeding behaviour in obesity. In this context, we tested the hypothesis that γ-oryzanol would alter DNA methylation status in the brain reward system, resulting in an attenuation of the preference for an HFD in mice.

## Methods

### Animals

Seven-week-old male C57BL/6J mice obtained from Charles River Laboratories Japan (Kanagawa, Japan) were housed (3–4 per cage) in specific-pathogen-free conditions at 24°C under a 12 h/12 h light/dark cycle. After a week of acclimatisation, 8-week-old mice were weight-matched and divided into two or three groups to undergo each experiment. The mice were allowed free access to food and water. All animal experiments were approved by the Animal Experiment Ethics Committee of the University of the Ryukyus (Nos. 5352, 5718 and 5943).

### Administration of γ-oryzanol and 5-aza-2′-deoxycytidine

To evaluate the preference for the HFD, γ-oryzanol (Wako Pure Chemical Industries, Osaka, Japan) was administrated to 8-week-old mice by gavage during the food choice test as previously described [14, 17]. For the other experiments, an HFD (D12079B; Research Diets, New Brunswick, NJ, USA) containing 0.4% γ-oryzanol was manufactured as pellets. The components of the diet are shown in electronic supplementary material (ESM) Table 1. After 12 weeks of feeding, tissue was collected from the striatum and hypothalamus. The daily intake of γ-oryzanol, estimated from the mean food intake of the mice, was approximately 320 μg/g body weight. The doses of γ-oryzanol were determined as previously described [14]. The 5-aza-2′-deoxycytidine (5-aza-dC; Sigma-Aldrich, St Louis, MO, USA) was intraperitoneally injected (0.25 μg/g body weight) three times a week for 12 weeks [18].

### Estimation of preference for dietary fat

To evaluate preferences for dietary fat, food tests provided a choice between chow and HFD (D12450B and D12451; Research Diets) as previously described [14]. The components of diet are shown in ESM Table 1. Briefly, the mice were allowed free access to chow and HFD. Intakes of chow and HFD were measured weekly and analysed for changes in preference for dietary fat. HFD preference was calculated according to the formula: HFD preference = [(HFD intake/total food intake) × 100].

### Bisulphite sequencing for DNA methylation

DNA was purified using a DNeasy Blood & Tissue Kit (QIAGEN, Tokyo, Japan). The DNA solution was mixed with freshly prepared 3 mol/l NaOH, incubated at 37°C for 15 min and added to 5.3 mol/l urea, 1.7 mol/l sodium bisulphite and 4.9 mmol/l hydroquinone. The solution was subjected to 15 cycles of denaturation at 95°C for 30 s and incubation at 50°C for 15 min [19]. The bisulphite-treated DNA was purified using MinElute PCR Purification Kit (QIAGEN) and amplified by PCR using a KAPA HiFi HotStart Uracil+ ReadyMix PCR Kit (KAPA Biosystems, Woburn, MA, USA) and primers around the CpG site of the promoter region of D2R. The primer sequences were as follows: forward primer, 5′-GTAAGAATTGGTTGGTTGGAGTTAAAA-3′; reverse primer, 5′-ACCCTACCCTCTAAAACCACAACTAC-3′. Next, the adapter sequences were added and cleaned up using Agencourt AMPure XP (Beckman Coulter, Brea, CA, USA). Samples were then pooled and loaded onto a GS Junior (Roche Diagnostics, Tokyo, Japan) for sequencing according to the manufacturer’s protocol. The methylation level was expressed as the percentage of methylated cytosines in all cytosines residues.

### DNMT activity assay

The DNMT enzymatic activity assay was performed using an EpiQuik DNA Methyltransferase Activity/Inhibition Assay Kit (Epigentek Group, Brooklyn, NY, USA) and EPIgeneous Methyltransferase Assay kit (Cisbio Japan, Chiba, Japan) according to the manufacturer’s protocols.

To assess the inhibitory activity of each compound on DNA methylation, the formation of *S*-adenosyl-l-homocysteine (SAH) was measured in the presence of each compound (20 μmol/l for screening assays), *S*-adenosyl methionine (SAM; 10 μmol/l) and DNMT substrate (4 ng/μl) at 37°C for 90 min. To evaluate the Michaelis–Menten kinetics, DNMT1 (20 μmol/l) was incubated with γ-oryzanol, SAM (5 μmol/l) and the indicated concentration of poly dI-dC at 37°C for 90 min. DNMT3a (100 μmol/l) and DNMT3b (100 μmol/l) were incubated with γ-oryzanol, SAM (5 μmol/l) and the indicated concentration of poly dG·dC at 37°C for 120 min. The assays were performed in quadruplicate. Extracted protein (0.75 mg/ml) was incubated with SAM (5 μmol/l), poly dI-dC (5 μg/ml), and poly dG·dC (5 μg/ml) at 40°C for 120 min, and SAH formation was measured.

### Oestrogen-related receptor-γ activity assay

The potential antagonistic activity of γ-oryzanol on the oestrogen-related receptor-γ (ERRγ) was assessed using the Human Estrogen-Related Receptor Gamma Reporter Assay System (INDIGO Bioscience, State College, PA, USA) according to the manufacturer’s protocol. Briefly, non-human mammalian reporter cells constitutively expressing active ERRγ were exposed to the indicated concentrations of each compound for 24 h in triplicate.

### Western blotting

This was performed as previously described [20] with antibodies against D2R (1:500, rabbit), dopamine transporter (DAT; 1:500, rabbit), tyrosine hydroxylase (TH; 1:1000, rabbit) (AB5084P, AB1591P and AB152, Merck Millipore, Billerica, MA, USA), signal transducer and activator of transcription 3α (STAT3α; 1:1000, rabbit), DNMT1 (1:1000, rabbit), DNMT3a (1:1000, rabbit) (nos. 8768, 5032 and 3598; Cell Signaling Technology, Tokyo, Japan), DNMT3b (1 μg/ml, rabbit), ERRγ (1:1000, rabbit) and β-actin (1:10,000, mouse) (ab16049, ab128930 and ab6276; Abcam, Cambridge, MA, USA).

### Quantitative real-time PCR

Gene expression was examined as previously described [14]. mRNA levels were normalised to *Rn18s* (18S rRNA). The primer sets used for the quantitative real-time PCR analyses are summarised in ESM Table 2.

### Statistical analysis

Data are expressed as mean ± SEM. One-way ANOVA and repeated-measures ANOVA followed by multiple comparison tests (Bonferroni–Dunn method) were used where applicable. Student’s *t* test was used to analyse differences between two groups. Differences were considered significant at *p* < 0.05.

## Results

### Pharmacological inhibition of DNMTs by 5-aza-dC attenuated the preference for dietary fat in mice

**Fig. 1:**
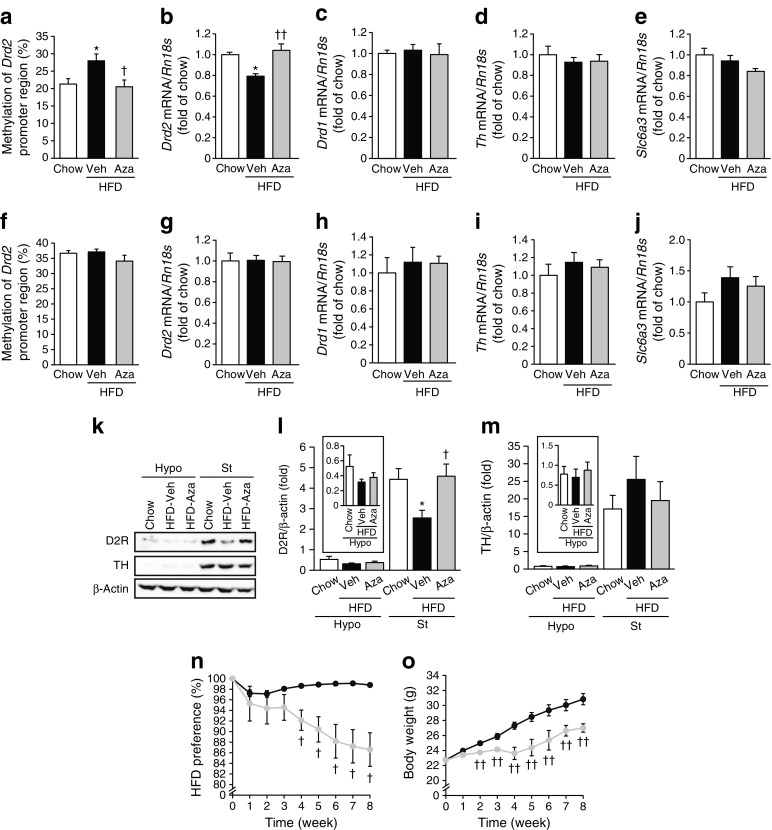
Inhibition of DNMTs by 5-aza-dC attenuates the preference for an HFD through augmentation of D2Rs in the striatum of HFD-fed mice. DNA methylation levels in the promoter region of D2R in the striatum (*n* = 3) (**a**) and hypothalamus (*n* = 3) (**f**). Levels of mRNA for *Drd2* (**b**, **g**), *Drd1* (**c**, **h**), *Th* (**d**, **i**) and *Slc6a3* (DAT) (**e**, **j**) in the striatum (*n* = 8) (**b**–**e**) and hypothalamus (*n* = 8) (**g**–**j**) of 5-aza-dC (Aza)-treated HFD-fed mice. Levels were normalised to those of *Rn18s*. Protein levels of D2R (**k**,**l**) and TH (**k**,**m**) in the hypothalamus (Hypo) and striatum (St) of 5-aza-dC-treated HFD-fed mice (*n* = 8); insets show values for hypothalamus plotted on a different scale. Protein levels were determined by western blotting. Values were normalised to those of β-actin. HFD preference (**n**) and body weight (**o**) in 5-aza-dC-treated mice during chow vs HFD choice tests (*n* = 4 cages; three mice per cage). Black circles, vehicle (Veh); grey circles, 5-aza-dC. Data are expressed as mean ± SEM. *p < 0.05, vs chow-fed mice; ^†^ *p* < 0.05, ^††^ *p* < 0.01 vs vehicle-treated HFD-fed mice

In mice fed the HFD, DNA methylation in the promoter region of D2R in the striatum was significantly augmented compared with mice fed a chow diet (Fig. 1a). On the other hand, hypothalamic DNA methylation in the promoter region of D2R was apparently higher than that in the striatum under a chow diet (*p* < 0.01) (Fig. 1a, f) and was not altered by the HFD (Fig. 1f). In mice fed the HFD, the augmented DNA methylation in the promoter region of D2R in the striatum was normalised by treatment with 5-aza-dC, a potent DNMT inhibitor (Fig. 1a). In contrast, DNA methylation in the promoter region of D2R in the hypothalamus was not significantly changed by treatment with 5-aza-dC (Fig. 1f). In the striatum of 20-week-old male mice fed the HFD for 12 weeks, mRNA and protein levels of D2R were significantly decreased (Fig. 1b, k, l). In contrast, levels of dopamine D1 receptors (D1Rs, encoded by *Drd1*), which act in an opposite manner to D2Rs on adenylyl cyclase and cAMP-mediated intracellular signalling, were unchanged (Fig. 1c). Furthermore, there was no change in the levels of other molecules related to D2R signalling, such as TH and DAT at the mRNA and/or protein level (Fig. 1d, e, k, m). On the other hand, no apparent changes were observed in the hypothalamus, including for D2R (Fig. 1g–m). Notably, protein levels of D2R and TH in hypothalamus were much lower than those in the striatum (Fig. 1l, m**)**, possibly reflecting the relative importance of dopamine receptor signalling in the brain reward system compared with the hypothalamus.

To examine whether DNA methylation in the promoter region of D2R would alter the preference for dietary fat, the feeding behaviour of 5-aza-dC-treated mice was analysed. As expected, 5-aza-dC significantly increased mRNA and protein levels for D2R in the striatum of HFD-fed mice (Fig. 1b, k, l). On the other hand, there was no effect on levels of *Drd1*, *Th* and *Slc6a3* (encoding DAT) in the striatum, or on levels of *Drd2*, *Drd1, Th* and *Slc6a3* in the hypothalamus (Fig. 1c–e, g–m). Whereas vehicle-treated mice preferred the HFD, preference for the HFD was significantly decreased in 5-aza-dC-treated mice (88% of the values for vehicle-treated mice) (Fig. 1n). Consequently, treatment with 5-aza-dC reduced the gain in body weight (Fig. 1o).

### γ-oryzanol decreases levels of DNMTs in striatum of HFD-fed mice

**Fig. 2:**
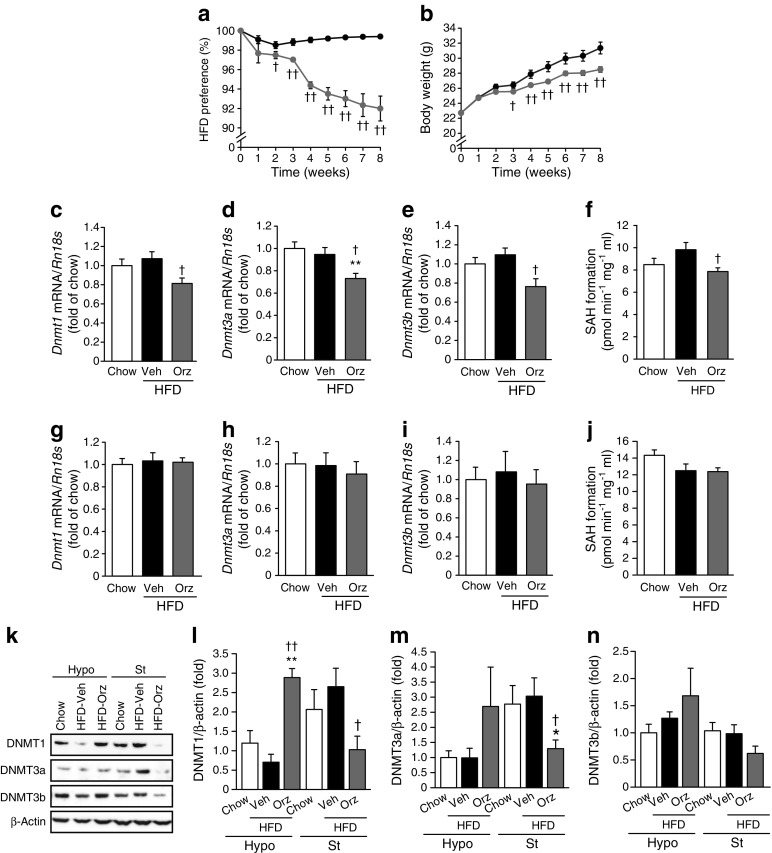
Inhibitory effect of γ-oryzanol on DNMTs in HFD-fed mice. HFD preference (**a**) and body weight (**b**) in γ-oryzanol-treated mice during food choice tests of chow vs HFD (*n* = 4 cages; three mice per cage). Levels of mRNA for *Dnmt1* (**c**, **g**), *Dnmt3a* (**d**, **h**) and *Dnmt3b* (**e**, **i**) in the striatum (*n* = 8) (**c**–**e**) and hypothalamus (*n* = 8) (**g**–**i**) of γ-oryzanol (Orz)-treated HFD-fed mice. Levels were normalised to those of *Rn18s*. Protein levels of DNMT1 (**k**, **l**), DNMT3a (**k**, **m**) and DNMT3b (**k**, **n**) in the hypothalamus (Hypo) and striatum (St) of γ-oryzanol-treated HFD-fed mice (*n* = 8). Protein levels were determined by western blotting. The same membrane was used as in Fig. 5k. Values were normalised to those of β-actin. Activities of DNMTs (measured by SAH formation) in the striatum (*n* = 7) (**f**) and hypothalamus (*n* = 7) (**j**) of γ-oryzanol-treated HFD-fed mice. Data are expressed as mean ± SEM. *p < 0.05, **p < 0.01, vs chow-fed mice; ^†^ *p* < 0.05, ^††^ *p* < 0.01 vs vehicle (Veh)-treated HFD-fed mice

As we previously reported [14], oral administration of γ-oryzanol to male mice by gavage significantly attenuated the preference for an HFD (93% of the values for vehicle-treated mice) (Fig. 2a), resulting in an apparent attenuation of body weight gain (Fig. 2b). We therefore explored the potential impact of γ-oryzanol on epigenetic modulation of D2Rs in the striatum.

In mammals, there are three major DNMTs—DNMT1, 3a and 3b. DNMT1 functions to maintain DNA methylation, while DNMT3a and 3b play a role in facilitating de novo DNA methylation [21]. To explore the potential impact of γ-oryzanol on DNMTs in vivo, we evaluated levels of DNMTs in the brains of HFD-fed mice. Although the HFD per se had no effect on mRNA and protein levels of DNMTs in either the striatum or the hypothalamus, supplementation with γ-oryzanol significantly decreased levels of DNMTs in the striatum but not in the hypothalamus (Fig. 2c–e, g–i, k–n). These data raise the possibility that γ-oryzanol might regulate levels of DNMTs in a striatum-specific manner. In a similar fashion, 5-aza-dC significantly decreased mRNA levels of DNMT3a and 3b preferentially in the striatum (ESM Fig. 1a–d).

**Fig. 3:**
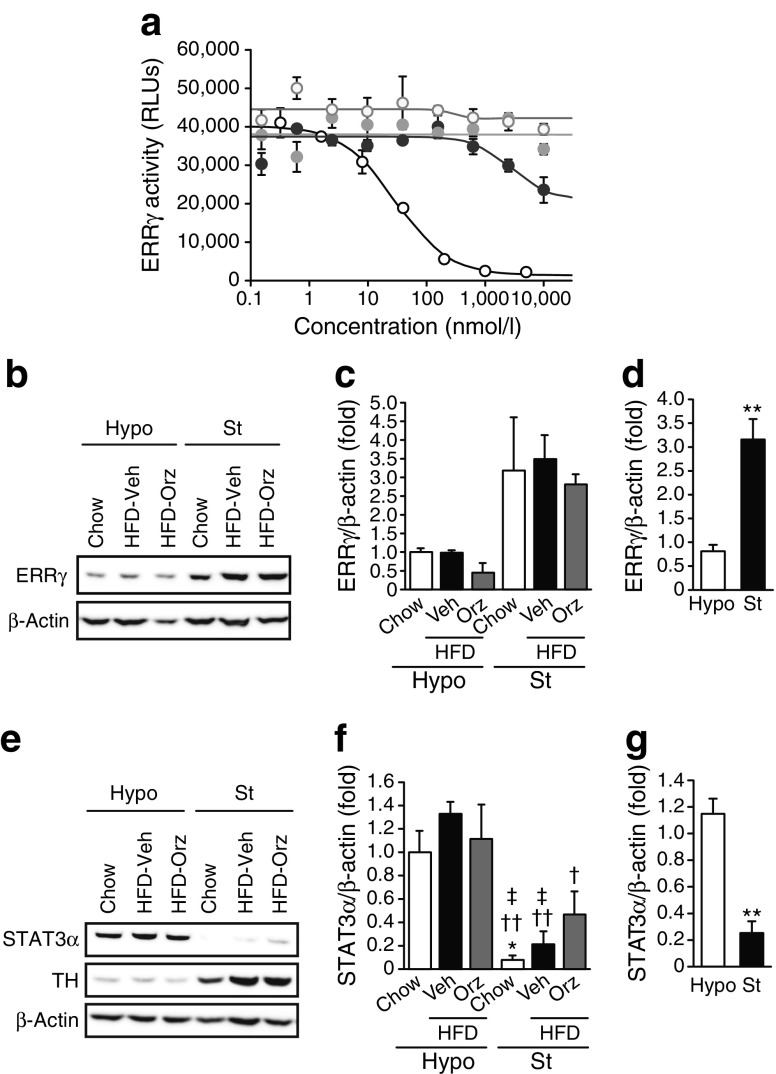
Impact of γ-oryzanol on ERRγ activity and STAT3α. (**a**) Inhibitory effect of γ-oryzanol on ERRγ in vitro. Dose–response curves of ERRγ activities with γ-oryzanol (black circles), ferulic acid (a metabolite of γ-oryzanol; grey circles), and 4-hydroxy tamoxifen (white circles with black outline), a potent inverse agonist for ERRγ. Activities of ERRγ are shown as relative luciferase units (RLUs). White circles with grey outline, 5-aza-dC. Levels of ERRγ (**b**–**d**), STAT3α (**e**–**g**) and TH (**e**) in the hypothalamus (Hypo) and striatum (St) of γ-oryzanol (Orz)-treated HFD-fed mice (*n* = 8). Protein levels were determined by western blotting. Values were normalised to those of β-actin. Data are expressed as mean ± SEM. *p < 0.05, **p < 0.01 vs hypothalamus from chow-fed mice or Hypo; ^†^ *p* < 0.05, ^††^ *p* < 0.01 vs hypothalamus from vehicle (Veh)-treated HFD-fed mice; ^‡^ *p* < 0.05 vs hypothalamus from γ-oryzanol-treated HFD-fed mice

On the basis of a previous study showing that the mRNA level of DNMT1 was positively regulated, at least partly, by the nuclear receptor ERRγ [22], we examined the potential effect of γ-oryzanol on ERRγ activity. In non-human mammalian cells constitutively expressing active ERRγ, 4-hydroxy tamoxifen, a potent inverse agonist of ERRγ, markedly decreased ERRγ activity. Of note, γ-oryzanol partially decreased ERRγ activity (an approximately 40% reduction of the innate value) (Fig. 3a). Importantly, ERRγ was highly expressed in the striatum but not in the hypothalamus (Fig. 3b–d). Contrary to the situation for the striatum, γ-oryzanol significantly increased protein levels of DNMT1 only in the hypothalamus (Fig. 2k, l). These results could be explained, at least partly, by our finding that STAT3α, a positive regulator of DNMT1 level [23], was abundantly expressed in the hypothalamus but not in the striatum (Fig. 3e–g).

To further assess the impact of γ-oryzanol on the activity of DNMTs in vivo, the formation of SAH, a byproduct of DNA methylation and also a potent inhibitor of DNMTs, was evaluated in γ-oryzanol-treated mice fed the HFD. There were no significant changes in SAH formation in either the striatum or the hypothalamus between HFD-fed and chow-fed mice (Fig. 2f, j). Noticeably, γ-oryzanol significantly decreased SAH formation in the striatum (Fig. 2f) but not in the hypothalamus (Fig. 2j), suggesting that γ-oryzanol may suppress the activity of DNMTs in a striatum-specific manner in HFD-fed mice.

### Enzymatic analyses on inhibitory properties of γ-oryzanol for DNMTs in vitro

**Fig. 4:**
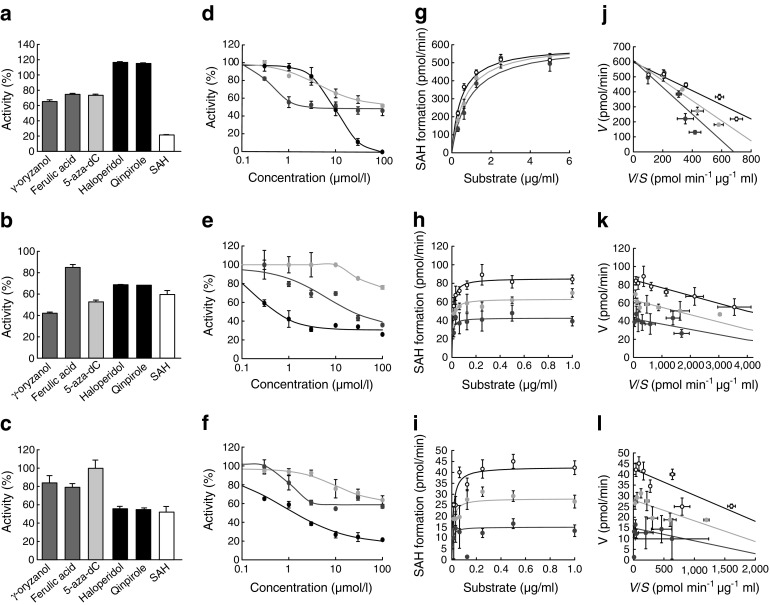
Inhibitory effect of γ-oryzanol on DNMTs in vitro. High-throughput screening assays for potential inhibitors of DNMT1 (**a**), DNMT3a (**b**) and DNMT3b (**c**). Inhibitory potentials against DNMTs for γ-oryzanol, ferulic acid (a metabolite of γ-oryzanol), 5-aza-dC, haloperidol (a D2R antagonist), quinpirole (a D2R agonist) and SAH (a well-documented potent inhibitor of DNMTs), were evaluated. Effects of various concentrations of γ-oryzanol on activities of DNMT1 (**d**), DNMT3a (**e**), and DNMT3b (**f**) are shown. Dark grey circles, γ-oryzanol; light grey circles, ferulic acid; black circles, SAH. Michaelis–Menten curves (**g**–**i**) and Eadie–Hofstee plots (**j**–**l**) for enzymatic inhibition against DNMT1 (20 μmol/l) (**g**, **j**), DNMT3a (100 μmol/l) (**h**, **k**), and DNMT3b (100 μmol/l) (**i**, **l**) in the presence or absence of γ-oryzanol. White circles, vehicle; light grey circles, 2 μmol/l γ-oryzanol; black circles, 20 μmol/l γ-oryzanol. Data are expressed as mean ± SEM. *V*, velocity (pmol/min); *V*/*S*, velocity/substrate concentration (pmol min^−1^ μg^−1^ ml)

We next assessed the impact of γ-oryzanol on the activity of DNMTs in vitro. The inhibitory potencies of γ-oryzanol, ferulic acid, 5-aza-dC, haloperidol (a representative D2R antagonist), quinpirole (a representative D2R agonist) and SAH against DNMTs were evaluated. As a positive control, SAH strongly attenuated the activities of DNMTs in a dose-dependent manner (Fig. 4a–f). As expected, haloperidol and quinpirole showed no effect on the activities of DNMTs (ESM Fig. 2). Noticeably, γ-oryzanol significantly inhibited the activities of DNMT1 (IC_50_ = 3.2 μmol/l), 3a (IC_50_ = 22.3 μmol/l) and 3b (maximum inhibition 57%) (Fig. 4d–f). In contrast, the inhibitory activity of ferulic acid, a metabolite of γ-oryzanol, was much lower than that of γ-oryzanol (Fig. 4d–f).

We further investigated the inhibitory properties of γ-oryzanol on DNMTs. The formation of SAH was measured to assess the inhibitory activity of γ-oryzanol on DNMTs in vitro. Data on SAH formation during DNMT-mediated DNA methylation indicate a saturable pattern of Michaelis–Menten kinetics for both the presence and absence of γ-oryzanol (Fig. 4g–i). In DNMT1-mediated DNA methylation, Eadie–Hofstee analysis demonstrated that γ-oryzanol showed no effects on the *V*_max_ of SAH formation (vehicle, 597 pmol/min; γ-oryzanol 2 μmol/l, 619 pmol/min; γ-oryzanol 20 μmol/l, 608 pmol/min), while γ-oryzanol apparently increased the *K*_m_ (vehicle, 0.47 μg/ml; γ-oryzanol 2 μmol/l, 0.67 μg/ml; γ-oryzanol 20 μmol/l, 0.89 μg/ml) (Fig. 4j). These results suggest that γ-oryzanol inhibits DNMT1 at least partly in a competitive manner. On the other hand, for DNMT3a- and 3b-mediated DNA methylation, γ-oryzanol decreased the *V*_max_ of formation of SAH (DNMT3a: vehicle, 85.3 pmol/min; γ-oryzanol 2 μmol/l, 63.1 pmol/min; γ-oryzanol 20 μmol/l, 42.5 pmol/min; DNMT3b: vehicle, 42.3 pmol/min; γ-oryzanol 2 μmol/l; 28.0 pmol/min, γ-oryzanol 20 μmol/l, 15.0 pmol/min) and, similarly, the *K*_m_ for this reaction (DNMT3a: vehicle, 0.0086 μg/ml; γ-oryzanol 2 μmol/l, 0.0080 μg/ml; γ-oryzanol 20 μmol/l, 0.0058 μg/ml; DNMT3b: vehicle, 0.0122 μg/ml; γ-oryzanol 2 μmol/l, 0.0097 μg/ml; γ-oryzanol 20 μmol/l, 0.0060 μg/ml) (Fig. 4k, l). These results suggest that γ-oryzanol inhibits DNMT3a and 3b at least partly in a non-competitive manner.

### γ-oryzanol increases levels of D2R in striatum of HFD-fed mice

**Fig. 5:**
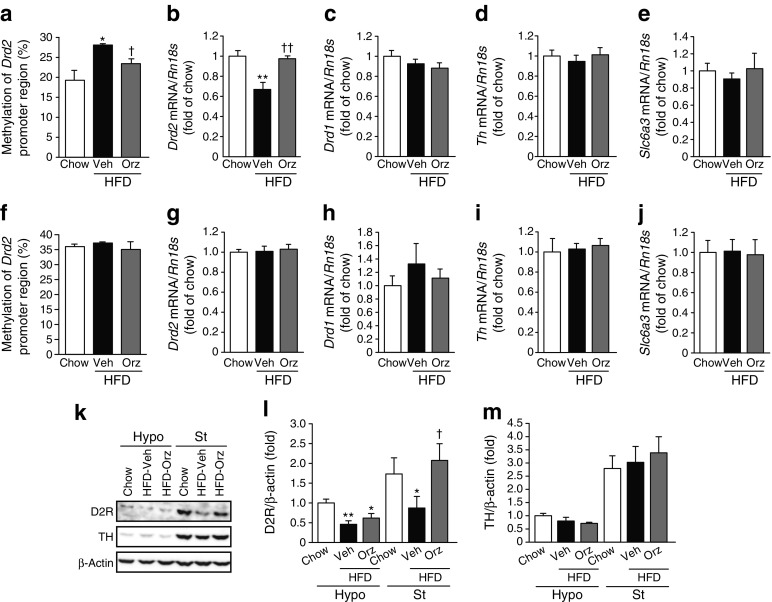
Inhibition of DNMTs by γ-oryzanol attenuates the preference for an HFD through augmentation of D2Rs in the striatum of HFD-fed mice. The DNA methylation levels of the promoter region of D2R in the striatum (*n* = 3) (**a**) and hypothalamus (*n* = 3) (**f**) of γ-oryzanol (Orz)-treated HFD-fed mice. Levels of mRNA for *Drd2* (**b**, **g**), *Drd1* (**c**, **h**), *Th* (**d**, **i**), and *Slc6a3* (DAT) (**e**, **j**) in the striatum (*n* = 8) (**b**–**e**) and hypothalamus (*n* = 8) (**g**–**j**) of γ-oryzanol-treated HFD-fed mice. Levels were normalised to those of *Rn18s*. Protein levels of D2R (**k**, **l**) and TH (**k**, **m**) in the hypothalamus (Hypo) and striatum (St) of γ-oryzanol-treated HFD-fed mice (*n* = 8). Protein levels were determined by western blotting. The same membrane was used as in Fig. 2k. Values were normalised to those of β-actin. Data are expressed as mean ± SEM. *p < 0.05, **p < 0.01 vs chow-fed mice; ^†^ *p* < 0.05, ^††^ *p* < 0.01 vs vehicle (Veh)-treated HFD-fed mice

We next tested the possibility that γ-oryzanol would increase striatal D2R content through an inhibition of DNMTs. In HFD-fed mice, oral administration of γ-oryzanol significantly decreased striatal DNA methylation in the promoter region of the D2Rs (Fig. 5a), whereas it did not do this in the hypothalamus (Fig. 5f). In accordance with these findings, mRNA and protein levels of D2R were reciprocally increased (Fig. 5b, g, k, l). Similar to the data on treatment with 5-aza-dC (Fig. 1), there were no apparent effects on the RNA and protein levels of *Drd1*, *Th* and *Slc6a3* (DAT) in the striatum, and no effects on levels of *Drd1*, *Th* and *Slc6a3* in the hypothalamus (Fig. 5c–e, h–k, m).

**Fig. 6:**
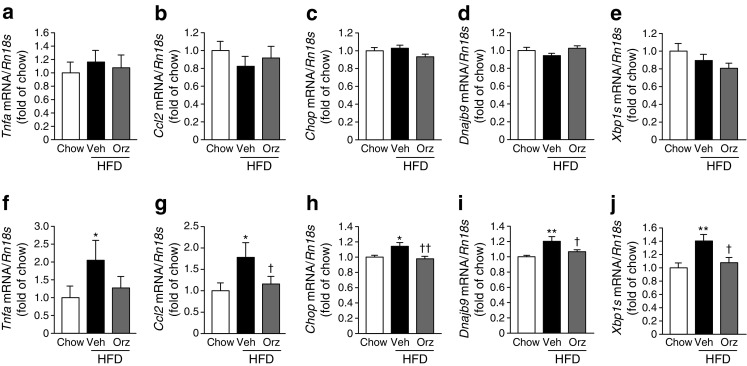
Expression of proinflammatory and ER stress-related genes in striatum and hypothalamus. Levels of mRNA for *Tnfa* (**a**, **f**), *Ccl2* (**b**, **g**), *Chop* (**c**, **h**), *Dnajb9* (**d**, **i**), and the active spliced form of *Xbp1* (*Xbp1s*) (**e**, **j**) in the striatum (*n* = 8) (**a**–**e**) and hypothalamus (*n* = 8) (**f**–**j**) of γ-oryzanol (Orz)-treated HFD-fed mice. *p < 0.05, **p < 0.01 vs chow-fed mice; ^†^ *p* < 0.05, ^††^ *p* < 0.01 vs vehicle (Veh)-treated HFD-fed mice. Data are expressed as mean ± SEM

Previous studies have shown that levels of D2R and DNMT1 are regulated by ER stress and inflammation at least partly via NF-κB [17, 24, 25]. We therefore examined the levels of ER stress-related and inflammation-related genes. As previously demonstrated [26], the HFD increased expression of the genes encoding TNF-α (*Tnfa*), monocyte chemoattractant protein–1 (MCP-1) (*Ccl2*), C/EBP homologous protein (*Chop*), ER-localised DnaJ 4 (ERdj4) (*Dnajb9*) and the spliced form of X-box binding protein 1 (*Xbp1s*) in the hypothalamus but not in the striatum (Fig. 6). Notably, supplementation of the HFD with γ-oryzanol significantly decreased the augmented expression of *Ccl2*, *Chop*, *Dnajb9* and *Xbp1s* exclusively in the hypothalamus but not in the striatum (Fig. 6).

## Discussion

The major finding in the present study is that γ-oryzanol acts as a potent DNMT inhibitor in the striatum of mice, thereby attenuating, at least partly, the preference for an HFD via the epigenetic modulation of striatal D2R. In striatum from HFD-fed mice, levels of D2R were significantly decreased, whereas those of D1R, TH and DAT were not changed (Fig. 1b–e, k–m). These data are consistent with the notion that dysregulation of striatal D2R plays a critical role in the perception of food reward when on an HFD, leading to hedonic overconsumption of HFD in obese animals [3]. In the present study, treatment of HFD-fed mice with 5-aza-dC significantly increased levels of striatal D2R (Fig. 1b, k, l) possibly through a reduction in DNA methylation level in the promoter region of D2R (Fig. 1a), and consequently attenuated the preference for dietary fat (Fig. 1n). This finding also supports a critical role of striatal D2Rs in the perception of food reward when on an HFD.

Our in vitro assay demonstrated that the inhibitory activity of γ-oryzanol against DNMTs was apparently stronger than that of its metabolite ferulic acid (Fig. 4d–f), suggesting the importance of the complete structure of γ-oryzanol for its inhibitory action on DNMTs. In HFD-fed mice, our studies suggest that, after oral administration, γ-oryzanol reaches the brain as a complete structure and decreases the levels and activities of DNMTs preferentially in the striatum, with a consequent decrease in DNA methylation in the promoter region of D2R in the striatum. Furthermore, our in vitro studies have demonstrated that γ-oryzanol acts as a partial antagonist against ERRγ, which primarily serves as a positive regulator for DNMT1 production [22], and consequently decreased the activity of DNMT1 (Fig. 3a). Of note, ERRγ was highly expressed in the striatum but not in the hypothalamus in mice (Fig. 3b). These data suggest that γ-oryzanol has the potential to decrease the mRNA level of DNMT1, at least partly, through the inhibition of ERRγ. In contrast to striatum, γ-oryzanol showed no effect on the level of D2R in hypothalamus from HFD-fed mice (Fig. 5g, k, l).

On the other hand, we demonstrated that γ-oryzanol significantly increased levels of DNMT1 in the hypothalamus but not in the striatum (Fig. 2k, l). It has been shown that STAT3 increases the content of DNMT1 in malignant T-lymphoma cells [23]. Notably, we previously demonstrated that γ-oryzanol significantly increased leptin-induced STAT3 phosphorylation in hypothalamus from HFD-fed mice [14]. It should also be noted that STAT3α was substantially expressed in the hypothalamus but not in the striatum in the mice (Fig. 3e–g). These data tempt us to speculate that the apparent difference in effect of γ-oryzanol on levels of DNMT1 between the hypothalamus and the striatum may be attributed, at least partly, to the region-specific content of STAT3α and ERRγ in the brain of mice (Fig. 3b–g). Collectively, there seems to be a reciprocal pattern of expression of ERRγ and STAT3α between the striatum and the hypothalamus in mice. On the basis of our results, it is therefore reasonable to speculate that in striatum, where ERRγ production is abundant, γ-oryzanol may preferentially decrease the mRNA level and enzyme activity of DNMT1 as a negative regulator of ERRγ. In contrast, in the hypothalamus, where STAT3α production is dominant, γ-oryzanol may preferentially increase levels of DNMT1.

A recent study demonstrated that an attenuation of striatal D2R signalling induced by an HFD dysregulates feeding behaviour [3], suggesting the potential importance of the inhibition of striatal DNMTs for the treatment of obesity. On the other hand, a previous study demonstrated a possibility that the status of DNA methylation of the melanocortin receptor 4 gene expressed in specific hypothalamic nuclei could modulate transgenerational forms of obesity in agouti viable yellow mice [27]. Although further studies are warranted to elucidate the underlying mechanisms, these studies suggest the importance of tissue-, gene- and sequence-specific DNA methylation in the pathophysiology of HFD-induced obesity.

We recently reported that HFD increased the level of D2R in the pancreatic islets of mice [17, 24]. It is likely that such augmentation is mediated, at least partly, by ER stress and inflammation via NF-κB, because there are several NF-κB-responsive elements in the promoter region of D2R [17, 24]. Furthermore, a recent study has shown that TNF-α and IL-1β increase the level and activity of DNMT1 in adipose tissue from HFD-fed mice [25]. Importantly, the present study demonstrated that HFD induced ER stress and inflammation preferentially in hypothalamus but not in the striatum (Fig. 6). In-depth mechanisms of tissue-, region-, and site- specific DNA methylation and demethylation in our experimental paradigm must await further investigation.

Together with our previous report showing that γ-oryzanol attenuates the preference for an HFD via hypothalamic regulation of ER stress in mice [14], γ-oryzanol also represents a unique property of ameliorating both hedonic and metabolic dysregulation of feeding behaviour. Because some antiobesity drugs that have been developed are known to cause critical adverse effects [8], a natural food-based approach toward the brain reward system is anticipated to treat obesity–diabetes syndrome safely [16]. In this paradigm, γ-oryzanol is a promising antiobesity candidate with a distinct property of being an epigenetic modulator.

## Electronic supplementary material

ESM (PDF 256 kb)

## Abbreviations

5-aza-dC: 5-aza-2′-deoxycytidine
D1R: Dopamine D1 receptor
D2R: Dopamine D2 receptor
DAT: Dopamine transporter
DNMT: DNA methyltransferase
ER: Endoplasmic reticulum
ERR: Oestrogen-related receptor
HFD: High-fat diet
SAH: *S*-Adenosyl-l-homocysteine
SAM: *S*-Adenosyl methionine
STAT3α: Signal transducer and activator of transcription 3α
TH: Tyrosine hydroxylase
