# Supplementary material for: Impact of brown rice-specific γ-oryzanol on epigenetic modulation of dopamine D2 receptors in brain striatum in high-fat-diet-induced obesity in mice
Source: Diabetologia. 2017 May 20;60(8):1502–11. doi: 10.1007/s00125-017-4305-4 (PMC5491592; doi:10.1007/s00125-017-4305-4)
Supplement: Supplementary file 1 — (PDF 256 kb) [file 125_2017_4305_MOESM1_ESM.pdf]

Electronic Supplementary Material (ESM) Information

ESM Table 1. Compositions of Chow and HFD

Component (g)	**chow D12450B**	**HFD D12451**	**HFD D12079B**
Casein	200	200	195
L-Cystine	3	3	
DL-Methionine			3
Com Starch	315	72.8	50
Maltdextrin 10	35	100	100
Sucrose	350	172.8	341
Cellulose	50	50	50
Soybeean Oil	25	25	
Lard	20	177.5	
Milk Fat, Anhydrous			200
Corn Oil			10
Mineral Mix S10026	10	10	
Mineral Mix S10001			35
DiCalcium Phosphate	13	13	
Calcium Carbonate	5.5	5.5	4
Potassium Citrate, 1 H2O	16.5	16.5	
Sodium Chloride	0	0	
Vitamin Mix V10001	10	10	10
Choline bitartrate	2	2	2
Total	1055.1	858.15	1001.54

Protein (kcal)	716	(18%)	716	(18%)	797 (17%)
Carbohydrate (kcal)	2856	(72%)	1438	(36%)	1968 (42%)
Fat (kcal)	405	(10%)	1823	(46%)	1921 (41%)
Total	3977		3977		4686

kcal/g	3.8	4.6	4.7

ESM table 2. The primer sets used for quantitative real-time PCR analysis

Gene	Primer (5′ to 3′)
Drd1	*f* ATG GCT CCT AAC ACT TCT ACC A
(D1R)	*r* GGG TAT TCC CTA AGA GAG TGG AC
Drd2	*f* ACC TGT CCT GGT ACGATG ATG
(D2R)	*r* GCA TGG CAT AGT AGT TGT AGT GG
Slc6a3	*f* GCA CTA CTT CTT CTC CTC CT
(DAT)	*r* CCT GAA GTC TTT ACT CCC TTC C
Th	*f* CCC TAC CAA GAT CAA ACC TAC C
(TH)	*r* GAG CGC ATG CAG TAG TAA GA
Dnmt1	*f* AAG AAT GTG TTG TCT ACC GAC
	r CAT CCA GGT TGC TCC CCT TG
Dnmt3a	*f* GAG GGA ACT GAG ACC CCA C
	r CTG GAA GGT GAG TCT TGG CA
Dnmt3b	*f* AGC GGG TAT GAG GAG TGC AT
	r GGG AGC ATC CTT CGT GTC TG
Ddit3	*f* CCA CCA CAC CTG AAA GCA GAA
(Chop)	*r* AGG TGA AAG GCA GGG ACT CA
Dnajb9	*f* CCC CAG TGT CAA ACT GTA CCA G
(ERdj4)	*r* AGC GTT TCC AAT TTT CCA TAA ATT
Xbp1s	*f* AGT TAA GAA CAC GCT TGG GAA TGG
	r CTG CTG CAG AGG TGC ACA TAG TC
Rn18s	*f* TTC TGG CCA ACG GTC TAG ACA AC
(18S rRNA)	*r* CCA GTG GTC TTG GTG TGC TGA

Forward and reverse primers are designated by *f* and *r*, respectively.

Sequences of TaqMan primers and probes

Gene	Primer (5′ to 3′)<br>Probe (FAM-5' -> 3'-TAMRA)
Tnf<br>(TNF-α)	*f* TCT CTT CAA GGG ACA AGG CTG<br>*r* ATA GCA AAT CGG CTG ACG GT<br>*p* CCC GAC TAC GTG CTC CTC ACC CA
Ccl2<br>(MCP-1)	*f* TTG GCT CAG CCA GAT GCA<br>*r* CCA GCC TAC TCA TTG GGA TCA<br>*p* CCC CAC TCA CCT GCT GCT ACT CAT TCA

Forward and reverse primers and probe are designated by *f*, *r*, and *p* respectively.

ESM Fig. 1. Inhibitory effect of 5-aza-dC on DNMTs in HFD-fed mice

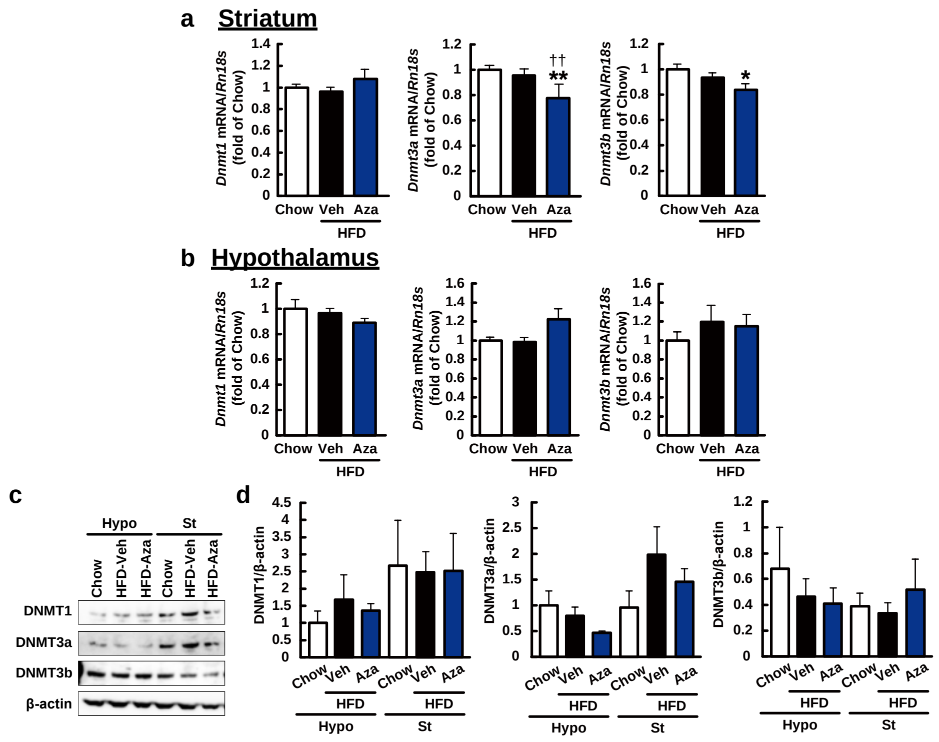

Level of mRNA expression for *Dnmt1*, *Dnmt3a* and *Dnmt3b* in striatum (n = 8) (**a**) and hypothalamus (n = 8) (**b**) in 5-aza-dC (Aza)-treated HFD-fed mice. The levels were normalized by those of *Rn18s*. (**c, d**) Protein levels of DNMT1, DNMT3a and DNMT3b in hypothalamus (Hypo) and striatum (St) from 5-aza-dC (Aza)-treated HFD-fed mice (n = 8). Protein levels were determined by Western blotting. Same membrane was analyzed as in Figure 1. The values were normalized by those of β-actin protein expression levels. *$P < 0.05$, **$P < 0.01$, *vs*. chow-fed mice (Chow). $^{\dagger}P < 0.05$, $^{\dagger\dagger}P < 0.01$ *vs*. vehicle (Veh)-treated HFD-fed mice. Data are expressed as means ± SEM.

ESM Fig. 2. DNMTs activity in the presence of haloperidol or quinpirole

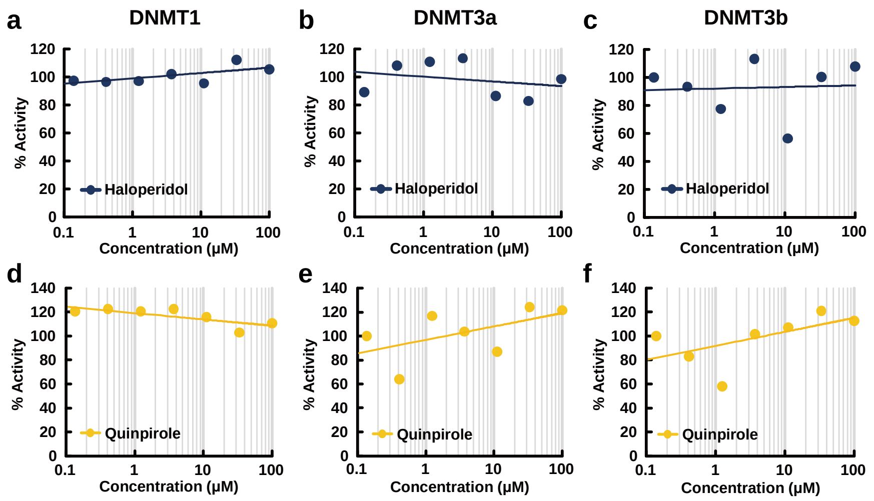

Effects of various concentrations of haloperidol (**a, b, c**) and quinpirole (**d, e, f**) on activities of DNMT1 (**a, d**), DNMT3a (**b, e**), and DNMT3b (**c, f**) were shown, respectively. The formation of SAH was measured. (**a, d**) DNMT1 (20 μM) was incubated with the indicated concentration of haloperidol or quinpirole, SAM (5 μM) and poly dI·dC (5 μg/ml) at 37°C for 90 min. (**b, c, e, f**) DNMT3a (100 μM) (**b, e**) and DNMT3b (100 μM) (**c, f**) were incubated with the indicated concentration of haloperidol or quinpirole, SAM (5 μM) and poly dG·dC (5 μg/ml) at 37°C for 120 min. The assays performed done in quadruplicate.
